# A Summary of Pain Locations and Neuropathic Patterns Extracted Automatically from Patient Self-Reported Sensation Drawings

**DOI:** 10.3390/ijerph22091456

**Published:** 2025-09-19

**Authors:** Andrew Bishara, Elisabetta de Rinaldis, Trisha F. Hue, Thomas Peterson, Jennifer Cummings, Abel Torres-Espin, Jeannie F. Bailey, Jeffrey C. Lotz, REACH Investigators

**Affiliations:** 1 Department of Anesthesia and Perioperative Care, University of California, San Francisco, CA 94143, USA; 2 Bakar Computational Health Sciences Institute, University of California, San Francisco, CA 94143, USA; 3 Department of Orthopaedic Surgery, University of California, San Francisco, CA 94143, USA; derinaldiselisabetta@gmail.com (E.d.R.); thomas.peterson@ucsf.edu (T.P.); jenncummings@ucsf.edu (J.C.); jeannie.bailey@ucsf.edu (J.F.B.); jeffrey.lotz@ucsf.edu (J.C.L.); 4 Research Unit of Orthopaedic and Trauma Surgery, Departmental Faculty of Medicine and Surgery, Universitá Campus Bio-Medico di Roma, 00128 Rome, Italy; 5 Department of Epidemiology & Biostatistics, University of California, San Francisco, CA 94143, USA; trisha.hue@ucsf.edu; 6 Department of Neurosurgery, University of California, San Francisco, CA 94143, USA; abel.torresespin@ucsf.edu; 7 School of Public Health Sciences, Faculty of Health Sciences, University of Waterloo, Waterloo, ON N2L 3G1, Canada

**Keywords:** chronic low back pain, body-map digitization, spatial pain phenotyping, automated image processing, digital health

## Abstract

**Background** Chronic low-back pain (LBP) is the largest contributor to disability worldwide, yet many assessments still reduce a complex, spatially distributed condition to a single 0–10 score. Body-map drawings capture location and extent of pain, but manual digitization is too slow and inconsistent for large studies or real-time telehealth. **Methods** Paper pain drawings from 332 adults in the multicenter COMEBACK study (four University of California sites, March 2021–June 2023) were scanned to PDFs. A Python pipeline automatically (i) rasterized PDF pages with *pdf2image* v1.17.0; (ii) resized each scan and delineated anterior/posterior regions of interest; (iii) registered patient silhouettes to a canonical high-resolution template using ORB key-points, Brute-Force Hamming matching, RANSAC inlier selection, and 3 × 3 projective homography implemented in OpenCV; (iv) removed template outlines via adaptive Gaussian thresholding, Canny edge detection, and 3 × 3 dilation, leaving only patient-drawn strokes; (v) produced binary masks for pain, numbness, and pins-and-needles, then stacked these across subjects to create pixel-frequency matrices; and (vi) normalized matrices with min–max scaling and rendered heat maps. RGB composites assigned distinct channels to each sensation, enabling intuitive visualization of overlapping symptom distributions and for future data analyses. **Results** Cohort-level maps replicated classic low-back pain hotspots over lumbar paraspinals, gluteal fold, and posterior thighs, while exposing less-recognized clusters along the lateral hip and lower abdomen. Neuropathic-leaning drawings displayed broader leg involvement than purely nociceptive patterns. **Conclusions** Our automated workflow converts pen-on-paper pain drawings into machine-readable digitized images and heat maps at the population scale, laying practical groundwork for spatially informed, precision management of chronic LBP.

## 1. Introduction

Chronic low-back pain (LBP) has emerged as the most significant single contributor to years lived with disability (YLDs) on a global scale, eclipsing every other specific disease entity tracked by the Global Burden of Disease consortium. In the 2016 analysis—which synthesized data from 195 countries and territories—investigators estimated that LBP was responsible for approximately 57.6 million years lived with disability (YLDs), representing 7.2 percent of all disability-adjusted life lived world-wide in that calendar year [1]. Put differently, nearly one in fourteen years that humans collectively spent living with health-related impairments in 2016 could be traced to chronic pain in the lumbar region. Epidemiologists caution that this figure is unlikely to plateau soon: demographic aging, longer life expectancy, and the rising prevalence of sedentary lifestyles mean the absolute burden associated with LBP is almost certain to climb further in the coming decades. Chronic pain disorders—spanning persistent low-back pain and other musculoskeletal conditions as well as neuropathic syndromes and cancer-related pain—place a staggering, long-term burden on individuals and health-care systems alike. Despite that reality, routine clinical evaluation in everyday practice is still often constrained to crude 0–10 numerical rating scales and broad, region-based body charts; these legacy tools were never designed to capture the fine-grained spatial patterns, evolving temporal dynamics, or rich qualitative descriptors (burning, shooting, throbbing, etc.) that clinicians often use to tailor interventions according to underlying pain mechanisms [2,3].

Pain body maps, the simple outlines on which patients shade or mark their painful regions, offer a fast, language-agnostic method to convert subjective discomfort into an objective visual record. By capturing both the location and surface area of pain in a single image, these maps help clinicians distinguish localized nociceptive pain from widespread or referred patterns, track spatial spread or regression over time, and communicate complex presentations across multidisciplinary teams without lengthy narrative notes. They also provide researchers with a low-cost, repeatable metric for phenotyping cohorts and quantifying treatment responses, making them a cornerstone of large epidemiological surveys and mechanism-based trials. Although the basic format has evolved, from Dürer’s early sketch through Palmer’s mid-twentieth-century patient drawings to today’s color-coded, pixel-precise digital templates, the core rationale remains unchanged: a well-constructed body map turns individual sensations into shareable data that underpin both clinical decision-making and translational research [4].

Following Hagedorn et al. [5], we turned hand-drawn body maps into calibrated, pixel-dense heat maps and analyzed them with modern machine learning. The calibration set, standardizing size, orientation, and intensity makes drawings from different visits and patients directly comparable. This conversion turns a subjective sketch into a reproducible, quantitative biomarker [6] that supports granular phenotyping of pain distributions. After prospective validation, the pipeline will be able to forecast individual trajectories, detect incipient pattern shifts, and automatically flag patients likely to deteriorate or fail first-line therapy, enabling earlier, targeted interventions.

In keeping with the artificial intelligence roadmap outlined by Hagedorn and colleagues [5], converting patients’ hand-drawn body-map sketches into calibrated, pixel-dense heat maps, and then processing those images with modern machine learning algorithms, transforms what was once a purely subjective drawing into a reproducible, quantitative biomarker [6]; this digital pipeline not only enables granular, objective phenotyping of pain distributions but also could enable longitudinal models to forecast individual symptom trajectories, identify incipient pattern shifts, and automatically flag patients who are most likely to deteriorate or fail first-line therapy at an early stage, pending prospective validation.

A rapidly expanding corpus of telehealth research now demonstrates that when detailed, digitized pain-map data are streamed into secure, cloud-based remote-care platforms, clinicians can visualize spatial pain trajectories in near real time. They can receive automated alerts when thresholds are crossed. They can consult decision-support algorithms that suggest dosage modifications, and immediately tailor pharmacotherapy, procedural scheduling, or rehabilitation regimens without waiting for the next face-to-face encounter. This capability is forecast to evolve from early-adopter innovation to everyday best practice across chronic pain services worldwide [7].

Automatically extracting rich, high-dimensional, machine-readable signatures from patients’ self-reported pain locations moves chronic pain management toward a true precision medicine framework: these digital fingerprints let clinicians fine-tune interventions to each individual’s spatial pain phenotype, enable telehealth platforms to deliver continuous, personalized monitoring at population-scale, and provide researchers with a standardized data layer that can be aggregated across institutions and countries for large collaborative studies and globally harmonized meta-analyses. Recent work shows that digital body maps can be captured repeatedly at scale and analyzed with modern pipelines—supporting high-frequency, multi-week monitoring and algorithmic conversion of freehand drawings into frequency/heat maps without grid constraints—and large network studies have validated region-based body maps as reliable measures of widespread pain, facilitating cross-cohort harmonization; our contribution operationalizes these advances into an end-to-end, open, automated pipeline for chronic low-back pain that yields ML-ready representations bridging research and real-world deployment [6,8,9,10].

We position this work within public-health measurement science: an open, low-cost, and language-agnostic pipeline that turns subjective drawings into standardized, population-ready spatial biomarkers for chronic pain, consistent with a transdisciplinary mission of health promotion and disease prevention.

## 2. Methods

### 2.1. Cohort Description

The drawings analyzed in the current spatial–phenotyping project were sourced exclusively from the University-of-California-San-Francisco-coordinated comeBACK cohort. COMEBACK (Chronic low-back pain Mechanisms and Biomarkers Across California) is an ongoing, prospective, multicenter observational study expressly designed to “deep-phenotype” adults living with chronic low-back pain by integrating self-reported symptoms, quantitative sensory testing, advanced imaging, digital pain maps, electronic health record data, and longitudinal patient-reported outcomes. The consortium comprises four University of California academic health centers—UCSF in San Francisco, UC Davis in Sacramento, UC Irvine in Orange County, and UC San Diego in La Jolla—thereby capturing the clinical and demographics that spans Northern, Central, and Southern California [11,12].

Recruitment opened in March 2021, targeting community advertisements, primary-care referrals, spine clinics, and electronic-health-record prescreening alerts. By the administrative cutoff of June 2023, the study had enrolled 450 adults, surpassing its original goal of 400, and enrollment remains open for selective sub-studies even as the main protocol transitions into the long-term follow-up phase. Eligibility criteria required an age of 18 to 75 years, a clinical history of low-back-dominant pain persisting for at least three months, and the ability to complete written and web-based questionnaires in English or Spanish; exclusion criteria ruled out red-flag spine conditions, major neurologic disorders, active cancer, or planned spine surgery within three months of screening.

Each participant attends an extensive baseline visit at their local UC site, during which research staff collect demographic data, medical history, comorbid symptom inventories, pain drawings, standardized photographs for posture analysis, quantitative sensory testing thresholds, and blood samples for biomarker discovery. Annual in-clinic reassessments at twelve and twenty-four months repeat these core evaluations and add spine MRI if clinically permissible. Between clinic visits, participants receive automated web links prompting monthly electronic surveys for the first six months, then quarterly surveys thereafter, with additional time points at months eighteen and twenty-four to capture intermediate changes. These instruments include numeric pain ratings, Oswestry Disability Index [13], PROMIS [14] scores, work productivity scales, and flare diaries.

To supplement direct reports, the protocol includes continuous passive surveillance through the UC Health data warehouse, allowing the coordinating center to pull imaging reports, procedure codes, medication dispensations, emergency-department encounters, and inpatient admissions in near real time. Study governance is overseen by an executive steering committee with site principal investigators, patient-advisor representatives, and biostatisticians who meet monthly to review recruitment, retention, and data-quality metrics. All procedures were reviewed and approved by WCG IRB under a single-site reliance model (IRB #20204648). Written informed consent was obtained from every participant before any research procedures, in alignment with the ethical principles of the Declaration of Helsinki and applicable federal regulations for human-subjects research. Together, these rigorous methodological safeguards ensure that the spatial body-mapping analysis presented here rests on a well-characterized, ethically conducted, and demographically diverse foundation.

### 2.2. Data Acquisition

Body-map assessments were administered as standardized five-page case-report booklets printed on paper and were given to participants during their baseline clinic visit. After completing demographic and clinical questionnaires on page 1, each participant was instructed to draw, with black pen provided at the visit, the precise areas where he or she felt pain, numbness, tingling, or pins-and-needles sensations. These sketches were made on pages 2, 3, and 4, which presented anterior and posterior male- and female-neutral silhouettes at a fixed scale so that the surface area would be comparable across subjects. Finished booklets were scanned. Immediately after upload, a data coordinator replaced all personal identifiers on the cover sheet with the study code, removed any stray patient notes that might reveal identity, and saved the de-identified PDFs into a read-only directory.

**Patient drawing instructions.** Participants were shown an example card and instructed to shade (not circle or annotate) every area that currently felt (a) pain, (b) numbness, or (c) pins-and-needles on the corresponding pages, using the black pen. Staff emphasized solid shading, avoiding text/marks outside silhouettes, and marking only present sensations.

### 2.3. Image Conversion

Prior to spatial analysis, a Python-based preprocessing script (version 3.10.17) iterated through the secure directory and programmatically opened each PDF. The script verified that the document contained exactly five pages, a rule that ensured every required drawing and questionnaire section had been completed. PDFs passing this validation step were parsed so that pages 2, 3, and 4 were individually exported as high-quality JPEG images to balance file size and fidelity. Filenames contained the study ID and the page of the PDF, which corresponds to either pain, numbness, or pins-and-needles.

### 2.4. Pre-Processing and Registration

**Figure 1:**
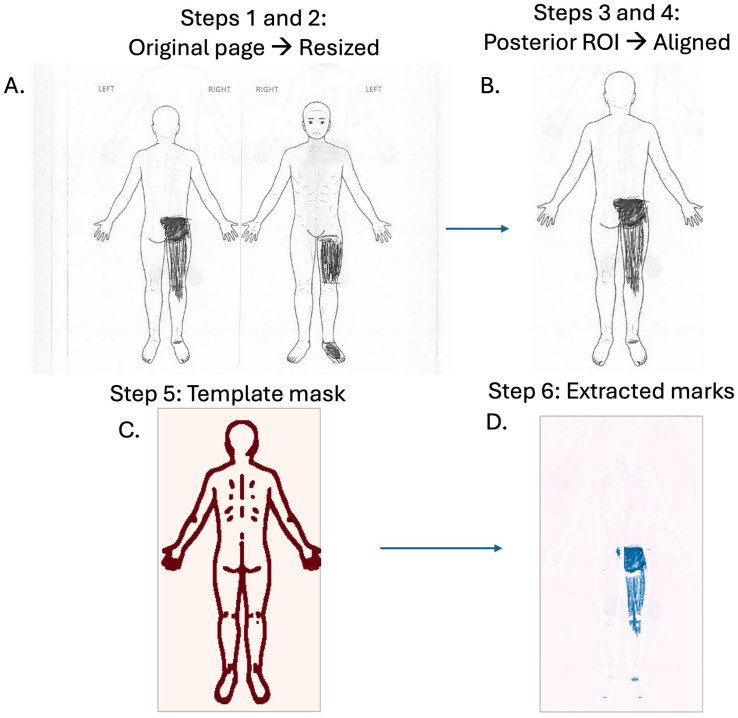
Key stages of the body-map processing pipeline. (**A**) Original patient page from the pain-drawing questionnaire, converted to grayscale and resized. (**B**) Posterior region of interest (ROI) extracted and aligned to a standardized template to correct for scale/rotation. (**C**) Template mask (red) defining the analyzable body surface; pixels outside the mask are excluded from downstream quantification. (**D**) Extracted patient marks (blue) after normalization and thresholding, used to compute regional coverage and heat maps. Arrows indicate processing flow (left→right, top→bottom); rounded frames are for visualization only.

A single, high-resolution blank body map served as the fixed reference template for all registrations. For every participant, the scanned image was first resized to match the template’s pixel dimensions, after which separate rectangular crops isolated the anterior and posterior torso regions of interest. Each cropped region was then individually aligned to the template through a feature-based registration pipeline. Specifically, ORB (Oriented FAST and Rotated BRIEF) keypoints and binary descriptors were computed on both the patient crop and the template; descriptor pairs were matched with a Brute-Force Hamming matcher [15]; putative correspondences were filtered with RANSAC to yield a robust inlier set; and the resulting inliers were used to estimate a 3 × 3 projective homography [16]. Finally, cv2.warpPerspective (opencv-python version 4.11.0) was applied to warp the participant’s region of interest into exact template coordinates using the homography. The procedure corrects differences in global scale, in-plane rotation, and small perspective distortions that can arise during hand scanning or photocopying [17]. An illustrative example of the alignment output is provided in Figure 1.

Registration accuracy was assessed using a proxy measurement approach. On a random 40-image subset (80 crops), alignment quality was quantified by the ORB/RANSAC inlier reprojection error in crop-pixel units. A quality control threshold of 3.00 px was established to trigger auto-reprocessing. A synthetic ground-truth test was performed using template crops with random perspective jitters (*N* = 80 synthetic warps across back and front) to validate the alignment approach.

### 2.5. Template Subtraction

**Template subtraction and binarization.** After registration, the reference ROI was converted to grayscale. Template lines were isolated with adaptive Gaussian thresholding (block = 35, C = 7) to highlight high-contrast edges across the silhouette despite lighting variation, and were then sharpened with a Canny detector (50/150) and dilated with a 3 × 3 kernel to guarantee full coverage of the printed outline [17]. A light Gaussian blur (σ ≈ 2) followed by a fixed threshold (0.41) thickened this mask. The mask was applied to the warped participant image, setting template pixels to background; subtracting it left only participant-drawn marks, which were then inverted so strokes appear solid black on white (Figure 1).

### 2.6. Quality Control

Every aligned region of interest was displayed in a tiled viewer alongside its subtraction output, and two independent team members manually inspected random cases for any leftover template traces, missing participant strokes, or evidence of misregistration. When problems were identified, the adaptive threshold parameters or the dilation kernel size were adjusted, and the subtraction procedure was rerun. This iterative tuning continued until accurate extraction of patient-drawn information was confirmed across the entire image set.

### 2.7. Symptom Descriptors and Pain Phenotype Categorization

Participants completed the sensory descriptor items from the painDETECT (PD-Q) questionnaire at the baseline: burning; tingling/prickling; pain with light touch (allodynia); sudden electric-shock–like pain; cold/heat-evoked pain; numbness; and pain with slight pressure (each 6-point Likert from “never” to “very strongly”). We used pre-specified definitions that map the sensory-item profile to nociceptive-leaning, neuropathic-leaning, nociplastic-leaning, or mixed categories [18,19].

### 2.8. Heat Map Construction

After template subtraction and quality control, every binary mask that passed inspection was resized to a uniform canvas so that all subsequent pixel operations referred to the same spatial grid. We then sorted the masks into six bins defined by sensation category—pain, numbness, and pins-and-needles—and by body orientation—anterior and posterior. Within each bin, all individual patient masks were stacked along a new axis to form a three-dimensional array, and a simple summation across that axis produced a two-dimensional density image whose pixel values represented the frequency with which a given point on the body was marked by participants. To make the densities comparable across sensations and view angles, the summed arrays were subjected to min–max normalization that linearly mapped the lowest observed count to 0 and the highest to 1. Finally, the normalized matrices were rendered with Matplotlib’s “hot” sequential color map, which encodes low densities in dark shades and high densities in bright yellows, yielding intuitive cohort-level heat maps for visual inspection and downstream quantitative analysis.

### 2.9. Composite Color Maps

**Figure 2:**
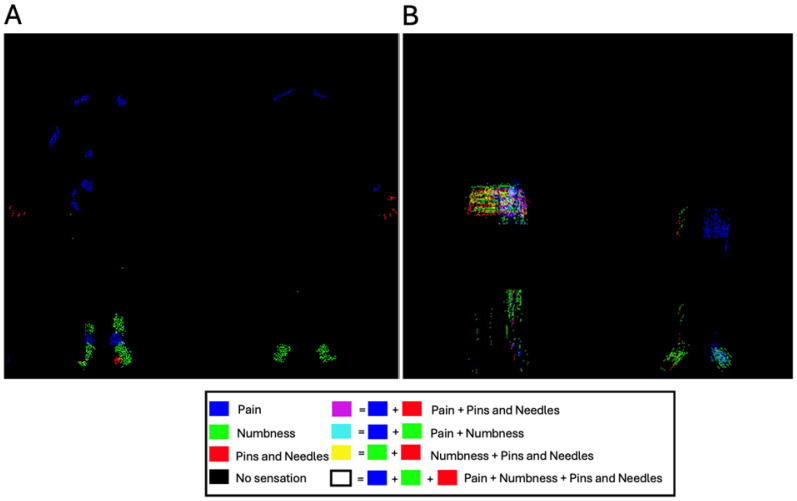
Composite RGB overlays for two participants. Red = pins-and-needles, green = numbness, blue = pain. Secondary colors indicate overlap (yellow = red + green, magenta = red + blue, cyan = green + blue; white = all three; black = none). Panels show anterior and posterior views for each participant; the legend summarizes color-to-sensation mapping. (**A**) Patient Example 1; (**B**) Patient Example 2.

For qualitative illustration, sensation-specific masks were assigned distinct RGB channels (pain = blue, numbness = green, pins-and-needles = red) and merged to generate patient-level composites. Composite images were saved for both anterior and posterior views. For qualitative visualization of overlapping symptom patterns, we converted the sensation-specific binary masks into separate red, green, and blue channels. Pixels corresponding to pain were loaded into the blue channel, pixels corresponding to numbness into the green channel, and pixels corresponding to pins-and-needles into the red channel. By merging these three single-channel images, we produced composite RGB overlays in which regions of mixed symptomatology appear as additive color blends; for example, locations coded as both pain and numbness display as yellow. Patient-level composites were generated for both anterior and posterior silhouettes, written to disk as lossless PNG files, and linked back to each participant’s study identifier for easy retrieval in electronic dashboards. See Figure 2 as an example.

### 2.10. Software Environment

All image-processing and visualization steps were scripted in Python 3.10.17 inside a reproducible Conda environment. PDF files were rasterized with pdf2image 1.17.0 (Poppler backend), after which OpenCV 4.11.0 handled feature extraction, homography estimation, warping, thresholding, and template subtraction. Pillow 11.2.1 provided high-level cropping and format conversion utilities, while NumPy 1.26.4 supported fast array operations such as stacking, summing, and normalization. Final static plots and interactive previews were produced with Matplotlib 3.10.3, ensuring that every figure in the manuscript could be regenerated end-to-end from the shared source code. Code was condensed and shared at https://github.com/ambishara/body_maps_ijerph (accessed on 13 August 2025).

### 2.11. Statistical Analysis

We summarized region of interest (ROI) level densities (lumbar paraspinal, gluteal, posterior thigh, lateral hip, lower abdomen) by phenotype group (nociceptive, neuropathic, nociplastic, mixed). Group contrasts used permutation tests (10,000 shuffles) with Benjamini–Hochberg FDR 0.05. Associations with age, BMI, and pain duration used Spearman ρ with FDR control. Cluster structure was explored with k-means on z-scored ROI vectors (k chosen by silhouette). We report standardized mean differences and 95% CIs.

## 3. Results

**Figure 3:**
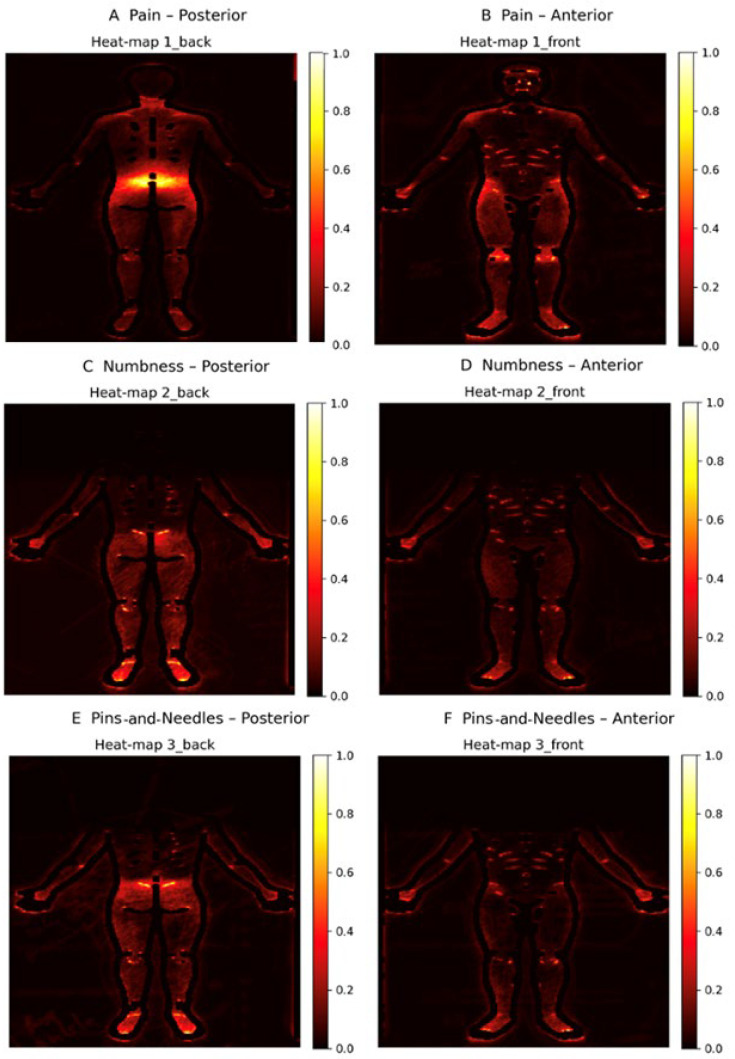
Population-level spatial distribution of patient-reported sensations (*N* = 332). Panels (**A**,**B**): pain (posterior, anterior); (**C**,**D**): numbness; (**E**,**F**): pins-and-needles. Heat maps show normalized frequency (0–1: brighter = more frequently marked across participants) within the template mask. Panel lettering, orientation labels, and color bars are consistent across rows.

**Figure 4:**
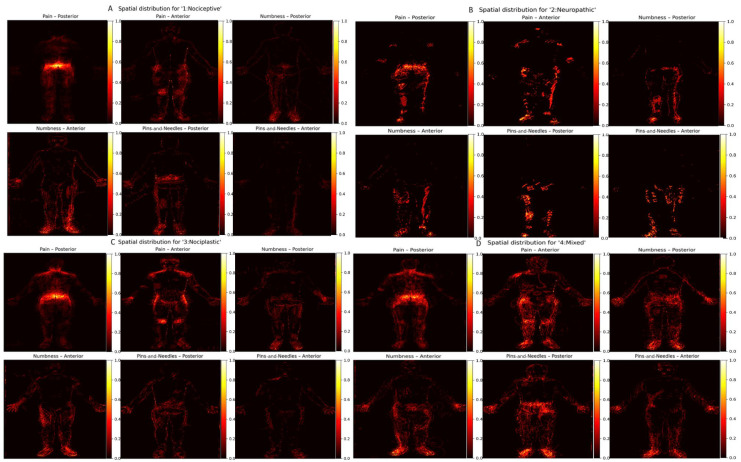
Spatial distributions stratified by pain phenotype for patients with pain phenotype determined (286/332). Cohort heat maps for nociceptive, neuropathic, nociplastic, and mixed groups are shown for posterior and anterior views (per-panel *N* indicated). Color scale reflects normalized frequency (0–1). Phenotype categorization was derived from a slightly modified version of the well-validated PainDETECT survey. (**A**) Spatial distribution for “1: Nociceptive”; (**B**) Spatial distribution for “2: Neuropathic”; (**C**) Spatial distribution for “3: Nociplastic”; (**D**) Spatial distribution for “4: Mixed”.

A total of 332 body maps were analyzed. Table 1 details the baseline characteristics of the analyzed sample, including age distribution, sex ratio, mean duration of chronic low-back pain, baseline numeric-rating scores, body-mass index, and current use of opioids or adjuvant analgesics. Figure 3 combines all accepted masks into a single cohort-level heat map, highlighting the ubiquitous hot spots over the lumbar paraspinal region, the gluteal fold, and the posterior thighs, while also revealing less commonly discussed clusters along the lateral hip and lower abdomen. Figure 4 stratifies these density surfaces by pain category, displaying separate panels for participants whose symptom descriptors suggested predominantly nociceptive, neuropathic, nociplastic, or mixed mechanisms; this side-by-side comparison highlights how neuropathic cases show more extensive leg involvement, whereas purely nociceptive cases remain tightly centered on the lumbar midline.

The median reprojection error was 0.96 px (IQR 0.57–1.53). No crops (0/80) exceeded the 3.00-px QC threshold, so no auto-reprocessing was triggered. Manual landmark-based TRE was not performed. In the synthetic ground-truth test, all alignment attempts were successful (successes = 80, failures = 0). The recovered alignment yielded a median grid-point error of 0.48 px (IQR 0.22–1.04; 95th percentile 3.01) and a median corner error of 1.31 px (IQR 0.65–2.49).

With regard to robustness, on a 50-image subset with an 80% subset bootstrap (B = 30), baseline variability was small but non-zero (MAE ≈ 0.003, r ≈ 0.96, bin-MAE ≈ 0.018, top-k Jaccard ≈ 0.67). One-dimensional sweeps near the defaults—block 25–45, C 5–9, Canny 40/120–60/180, dilation 3–5, stroke threshold 18–22, and post-blur thresholds 0.35–0.47—yielded near-identical cohort maps and preserved hotspot ranks. An aggressive blur reduction (σ = 1.5) modestly reduced similarity (r ≈ 0.84), so the default settings were retained.

ROI densities were analyzed for *N* = 286 subjects who had pain classification specified (nociceptive *n* = 127, neuropathic *n* = 12, nociplastic *n* = 81, mixed *n* = 66). Across ROIs, BH-FDR (q = 0.05) yielded 0 significant pairwise contrasts and 1 significant ROI–covariate association. The most pronounced contrasts included the fact that no pairwise differences survived BH-FDR q ≤ 0.05. Significant associations included lumbar_paraspinal~age: ρ = −0.23, q = 0.001, and *n* = 279. k-means on z-scored ROI vectors suggested k = 2 (silhouette = 0.554; cluster sizes: C0 *n* = 243, C1 *n* = 43).

## 4. Discussion

This proof-of-concept investigation demonstrates that a completely automated, fully open-source pipeline can take pen-on-paper pain drawings gathered from a multi-site clinical-trial cohort and convert them into anatomy-preserving, quantitative heat maps in less than one second for each scanned image, even when running on commodity laptop hardware. Earlier approaches to body-map digitization often required labor-intensive manual tracing with a stylus, mouse-driven masking inside Photoshop, or other semi-automated steps that introduced human variability, slowed throughput, and discouraged routine use in large studies. In contrast, our workflow performs three critical operations in a single script: first, it uses an ORB-feature detector [15] followed by homography [16] estimation to register every patient silhouette onto a common canonical template, thereby preserving anatomic proportions and permitting pixel-wise comparison across individuals; second, it removes guideline artifacts such as text labels, arrows, and calibration grids through template subtraction and morphological filtering, leaving only the patient’s inked pain regions; third, it stacks the cleaned binary masks from all subjects into high-resolution density surfaces that can be ingested directly by convolutional neural networks, transformer encoders, or other machine-learning architectures without additional preprocessing.

The composite maps we produced, illustrated in Figure 3 and Figure 4, are consistent with the classic topography of chronic low-back pain, including hotspots over the lumbar paraspinal muscles, the gluteal fold, and the posterior thigh. At the same time, the higher spatial resolution reveals under-appreciated clusters of reported numbness and paresthesia along the lateral aspect of the hip, a finding that may reflect peripheral entrapment or radicular involvement. Such detail is virtually impossible to capture with a single 0–10 numeric rating scale, yet it is exactly the kind of granularity required for modern mechanism-based phenotyping that differentiates nociceptive, neuropathic, and nociplastic components and thereby guides more targeted interventions. These results fit within and extend prior body-map quantification work—including standardized instruments such as the Michigan Body Map and newer computational pipelines—and align with evidence that body-map topography indexes sensory-processing phenotypes [6] to facilitate reuse and benchmarking across cohorts [3,8,10,20].

**Clinical workflow integration.** In practice, these maps fit naturally into remote-care dashboards and EHR side-panels: (i) medication titration—a weekly threshold alert for new anterior thigh spread triggers a best-practice card to adjust neuropathic adjuvants and schedule follow-up; (ii) interventional triage—a persistent posterior-leg cluster with dermatomal spread launches a checklist for radicular work-up and fast-track referral; (iii) rehabilitation personalization—lateral-hip clusters auto-populate targeted gluteal and hip-abductor protocols; and (iv) post-procedure monitoring—post-epidural steroid injection regression of the posterior-leg heat map below a predefined delta suppresses routine visits while flare detection re-opens access. Each scenario uses a fixed-size tensor → rules/ML → human confirmation pathway to keep clinicians in the loop.

From a telehealth and implementation perspective, the fact that drawings collected at four separate University of California sites could be standardized within seconds and processed in real time on everyday computers suggests that continuous remote monitoring of spatial pain patterns is technically and economically feasible at the population scale. Because the final output for each patient is a fixed-size pixel tensor, the data can be streamed through secure APIs into dashboard visualizers, longitudinal forecasting models, or decision-support tools that triage patients toward medication adjustments, advanced interventional procedures, or rehabilitative programs without an in-person visit. The same tensor can be archived in electronic health records and aggregated across institutions for large meta-analyses, opening the door to globally harmonized research on spatial pain phenotypes.

Several limitations should be noted. First, the drawings are self-reported and encode presence or absence of pain at each location but do not capture intensity gradients, temporal fluctuations, or sensory qualities such as burning versus stabbing. We encoded presence/absence rather than intensity because the paper form lacked calibrated severity markers, and ink density is confounded by pen pressure and scanning. The forthcoming digital version will add calibrated sliders and per-region intensity fields to enable weighted heat maps in future analyses. Second, the study focused only on chronic low-back pain, so generalizability to multisite pain conditions, inflammatory disorders, or pediatric populations remains to be established. Third, the present analysis is cross-sectional; future work must determine whether these heat map signatures remain stable over months, correlate with functional outcomes, and predict response to therapies. Even with these constraints, the pipeline provides a straightforward, high-yield method for collecting extremely granular spatial data from large numbers of patients and doing so in a way that scales effortlessly from single clinics to integrated health-system networks.

**Longitudinal roadmap (planned analysis).** This report is intentionally cross-sectional; no longitudinal results are included. In a preregistered follow-on analysis, we will evaluate (i) stability (test–retest similarity of regional densities at ~6, 12, and 24 months), (ii) prognostic value (whether baseline spatial features predict ODI and PROMIS change), and (iii) treatment response (within-person shifts after ESI, targeted PT, or opioid de-escalation). Analyses will use region-of-interest (ROI) level effect sizes with permutation-based *p*-values and Benjamini–Hochberg (BH) false discovery rate (FDR) control; endpoints and model families will be specified a priori.

## 5. Conclusions

Automated digitization of paper-based pain body maps transforms what was once a hand-drawn, subjective sketch into a quantitative, pixel-level biomarker that can be stored in electronic health records, piped directly into machine-learning pipelines, and analyzed alongside physiological or psychosocial covariates. The resulting data set captures nuanced spatial details such as total affected surface area, laterality, and multi-region overlap, giving predictive algorithms information they simply cannot extract from a single 0–10 pain score. These same high-resolution maps may help guide mechanism-informed care and could support near-real-time dashboards, pending validation. By demonstrating rapid, fully automated processing across a large cohort of adults with chronic low-back pain, the present work establishes both technical feasibility and clinical relevance, creating a clear pathway for embedding spatial pain signatures into precision-pain platforms, electronic consults, and eventually the everyday workflows of multidisciplinary pain clinics.

## Figures and Tables

**Table 1:** Baseline characteristics of the analyzed cohort (*N* = 332). Values are mean ± SD or %, as noted. “Missing/Unknown” indicates absent demographic data at baseline. See Methods for inclusion criteria and the Results opening paragraph for N reconciliation.

	Full Analysis Cohort
***N***	332
Age, years (mean ± SD)	54.17 ± 16.26
Body Mass Index (BMI), kg/m^2^ (mean ± SD)	26.64 ± 4.94
Sex (%)	
- *Male*	40.06
- *Female*	56.33
- *Unknown/Missing*	3.61
Race (%)	
- *Asian*	10.84
- *White*	71.39
- *Black or African American*	6.33
- *Native Hawaiian or Other Pacific Islander*	0.90
- *Mixed Ethnicity*	6.93
- *Missing*	3.61
Education Level (%)	
- *Doctoral or Postgraduate Education*	39.46
- *College or Bachelor’s Degree*	39.76
- *Associate or Technical Degree*	9.04
- *High School (Secondary) Degree*	7.83
- *Did not complete secondary education*	0.30
- *Missing*	3.61

## Data Availability

The data that support the findings of this study are openly available in the Vivli repository at: https://doi.org/10.25934/PR00010819 (accessed on 1 September 2025).
